# The Synergistic Effects of the Micro and Nano Particles in Micro-nano Composites on Enhancing the Resistance to Electrical Tree Degradation

**DOI:** 10.1038/s41598-017-08761-w

**Published:** 2017-08-17

**Authors:** Wenxuan Wang, Ying Yang

**Affiliations:** Department of Electrical Engineering, Tsinghua University, Beijing, 100084 China

## Abstract

A new method of increasing the durability and reliability of polymer dielectrics has been proposed by designing a composite structure of the micro and nano particles. The synergistic effects of the micro particles and nano particles are found to enhance the resistance to electrical tree and extend the lifetime of polymer dielectrics for insulations. Epoxy loaded with the micro and nano SiO2 particles at different concentrations are prepared as micro-nano composites. The micro particles show the blocking effects on the electrical tree channel and the interfaces of the nano particles lead to the inhibiting effects on the tree inception and propagation. The lifetime of the micro-nano composite samples in the experiments extends to 4 times of the neat epoxy. The new type of micro-nano composites can be widely applied in future electronic and electrical energy areas.

## Introduction

The reliability of electrical equipment and electronic devices becomes a big issue right now since once the insulating materials break down, the equipment can no longer serve on line further more. The durability of the polymer dielectrics is essential to the lifetime of the electrical equipment and electronic devices such as capacitors, batteries and cables^1–4^. Polymer degradation and electrical tree growth arise from the creation of intense electric fields at the tips of conducting inhomogeneity, discharge channels and sites of stress concentration^5^. The intense electric field leads to localized breakdown, creating cavities and tree channels^6^ which influence the long term degradation of the polymer dielectrics. If the growth these electrical tree could be avoided, the lifetime and reliability of the insulation could be greatly increased.

Using fillers to enhance the performance of polymer dielectrics has attracted widespread attention^7, 8^. High concentration (50 wt%) of micro inorganic particles are first applied in improve the resistance to the electrical tree^6, 9–12^. However, the high loading of the micro particles usually causes the insulation problems such as increased dielectric loss and decreased breakdown strength^13^. Recently, similar improvements of the resistance have been found using less amount (<10 wt%) of the nano particles in many researches. The nano particles are believed to expand the width of the electrical tree^14, 15^, inhibit the charges injection^16, 17^, capture the mobile charges with deep traps^18–20^, act against the partial discharge erosion^21–23^ and enhance the bonding strength of the polymer^24^.

In this paper, the big volume of the micro particles and the large surface area of the nano particles are both found to contribute to enhancing the resistance to electrical tree. Although there are many researches on the electrical tree behaviors of the micro composites, nano composites and the comparison of two, few has investigated into the resistance to electrical tree of the micro-nano composites containing both micro and nano particles. A feasible way to combine the advantages of both micro and nano particles is to design a structure which has not only big volume but also large surface area. However, these two features are usually contradictory for traditional particle structures. Here, a composite structure of micro particle covered by nano particles is proposed to have the two advantages. This structure can be achieved by blending surface modified micro and nano particles in solvent. Epoxy/SiO_2_ composites with certain proportions of micro/nano particles are tested to evaluate the resistance to electrical tree breakdown. The dielectric parameters are measured to study the interfacial polarizations of the composites which are related to the effects of the nano particles. The electrical tree growth in the composites with low particles concentrations (<1 wt%) are observed under a stereomicroscope to study the effects of the micro particles on enhancing resistance to electrical tree. The synergistic effects of the micro and nano particles are proposed as well as the blocking effects of the micro particles and the inhibiting effects of the nano particles, which explain the high performance of the micro-nano composites for long-term electrical insulations.

## Results

To study the properties of the micro-nano composites, epoxy/SiO2 composites with different total SiO2 concentrations and different micro/nano proportions were prepared for the followed experiments. In this paper, the neat epoxy resin samples are labelled as EP, and epoxy/SiO2 composites are labelled as CX-X/X. For example, C10-1/3 represents the composite with a total SiO2 concentration of 10 wt%, while the micro/nano proportion is 1/3, which means there are 2.5 wt% micro SiO2 and 7.5 wt% nano SiO2 in the composite. The incorporation of SiO2 can be seen from the TGA curves and NMR spectrums (Supplementary Information 1).

### SEM images of the composites

**Figure 1:**
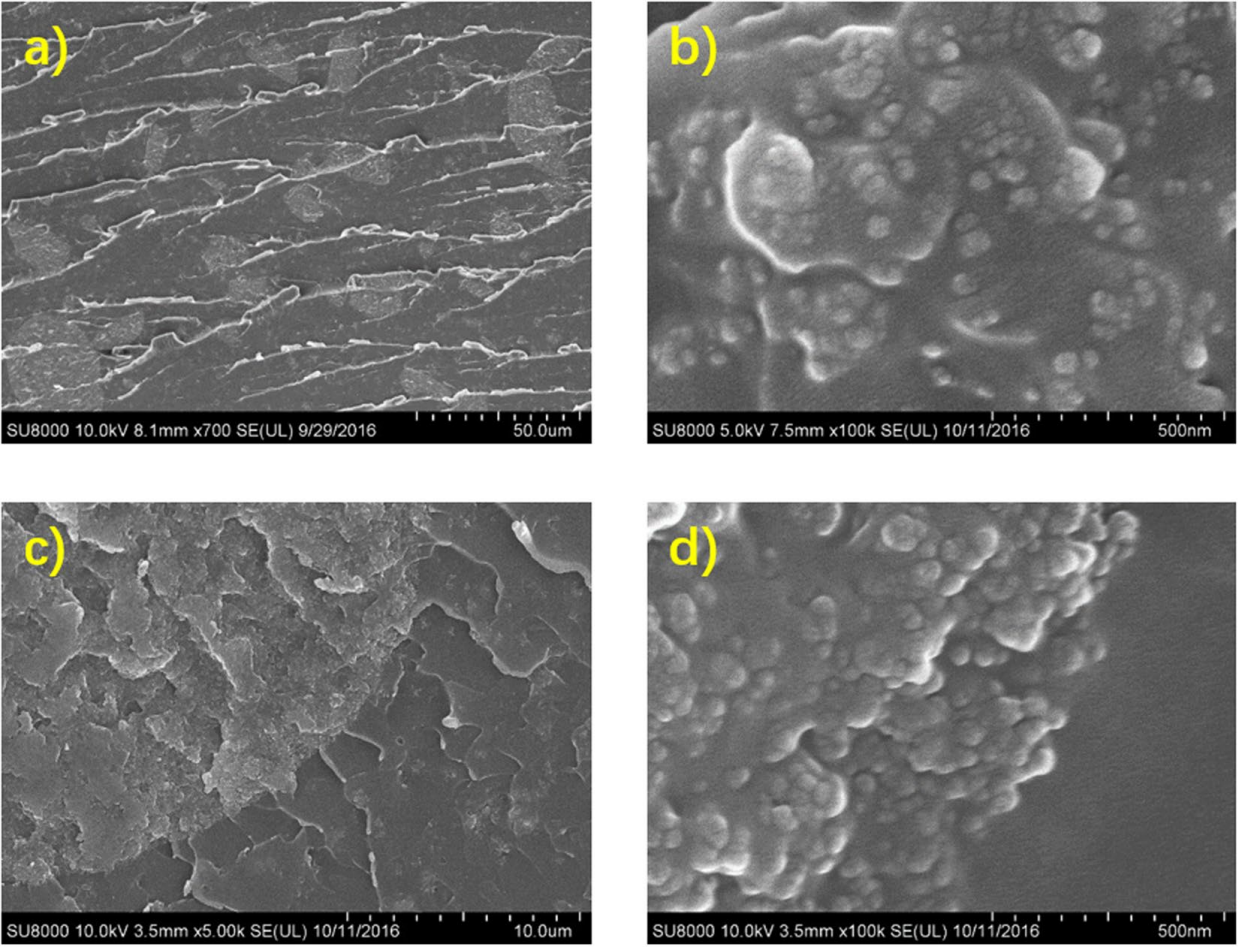
Particle dispersion in (**a**) the micro composite and (**b**) the nano composite. (**c**) A quarter of one micro SiO2 particle in the micro-nano composite. (**d**) The nano SiO2 particles on the surface of the micro SiO2 particle in the micro-nano composite.

The particle dispersions of the micro and nano particles in the micro and nano composites are shown in Fig. 1a and b. The size of the micro particles is 10~30 um and the size of the nano particles is 25~50 nm, which almost match the labeled size of 20 um and 30 nm. Figure 1c show a quarter of one micro SiO2 particle in the micro-nano composite, along with the nano particles in the composite on the bottom right. When the edge of the micro particle is zoomed in, the composite structure is found to be the micro particle covered by the nano particles as shown in Fig. 1d.

### Electrical tree breakdown tests

**Figure 2:**
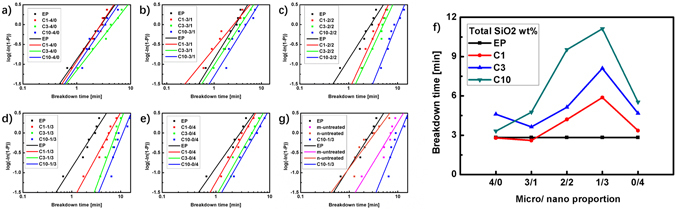
(**a–e**) Weibull distribution of the breakdown time of the epoxy/SiO2 composites with different total SiO2 concentrations and different micro/nano proportions. (**f**) The scale parameter $\alpha$ that indicates the breakdown time at the cumulative probability of 63.2%. (**g**) Weibull distribution of the breakdown time of the epoxy/SiO2 composites with a total SiO2 concentration of 10 wt% and a micro/nano proportion of 1/3.

The resistance to electrical tree of the composite material can be directly assessed by the breakdown time during the electrical treeing experiment under the same electrical aging conditions. It is generally recognized that the breakdown probability of a dielectric obeys the Weibull distribution^25, 26^. Figure 2a to e shows the Weibull distribution (Supplementary Information 2) of the breakdown time of the epoxy/SiO2 composites. The Weibull distribution scale parameter $\alpha$ obtained from these figures are plotted in Fig. 2f.

Generally, in a Weibull distribution, one line on the right of the other line means it has a higher scale parameter $\alpha$ than the other. As shown in Fig. 2a, the breakdown time of CX-4/0 with only micro SiO2 first increases then decreases with SiO2 loading, which is similar with previous research on the micro Al2O3 particles filling^27^. The particles in dielectrics are believed to block the electrical tree channel: when the tree reaches the particles, it will grow transversely to bypass the particles because it is hard for the tree to grow through them^24, 28, 29^. This kind of bypass will not only consume more energy but also increase the width of the electrical tree, both of which lead to the slower tree growth and the longer breakdown time. However, the micro particles have also been found to introduce defects into the composites^30, 31^. Therefore, when the concentration of the micro particles is high enough, the electrical tree will grow along these defects on the surface of the particles easily and thus reduce the breakdown time of epoxy.

As for CX-0/4 with only nano SiO2 shown in Fig. 2e, it can be clearly seen that incorporated particles have a positive role on the breakdown time of the composites in the whole investigation mass range. Many different theories have been proposed to explain the effects of the nano particles on inhibiting the development of the electrical tree. Besides the blocking effects mentioned above, most of these theories are essentially related to the interface between the nano particles and the polymer matrices. The interface area of the nano particle is much larger than that of the micro particle, so the interface dominates the properties of the nano composites^32, 33^. In the investigation mass range no more than 10 wt%, the interface area increases with higher concentration of the nano particles, which will enhance the inhibiting effects of the interface and thus increase the breakdown time of the composites.

By mixing both micro and nano particles into epoxy resin, composites with longer breakdown time are obtained as shown in Fig. 2f. As a control, the breakdown time of EP is 2.8 min. For the composites with only micro/nano SiO2, C3-4/0 and C10-0/4 have the longest breakdown time of 4.6 min and 5.5 min respectively. Among all the composites, C10-1/3 has the longest breakdown time of 11.1 min which is about 4 times of that of EP. The micro SiO2 concentration of C10-1/3 is 2.5 wt% which is close to the 3 wt% of C3-4/0, so the micro SiO2 in C10-1/3 should also positively affect the breakdown time. It can be inferred that the blocking effects of the micro particles and the inhibiting effects of the nano particles both exist in the micro-nano composites.

As shown in Fig. 2g, when the micro/nano particles used in C10-1/3 are replaced with untreated particles, the breakdown time is not as long as the both treated samples of C10-1/3. As for the samples filled with untreated nano particles, the Weibull breakdown time is 3.4 min, which is much closer to that of EP. It shows that the surface modification is much more important for the nano particles in the composites.

### Dielectric parameters measurement

**Figure 3:**
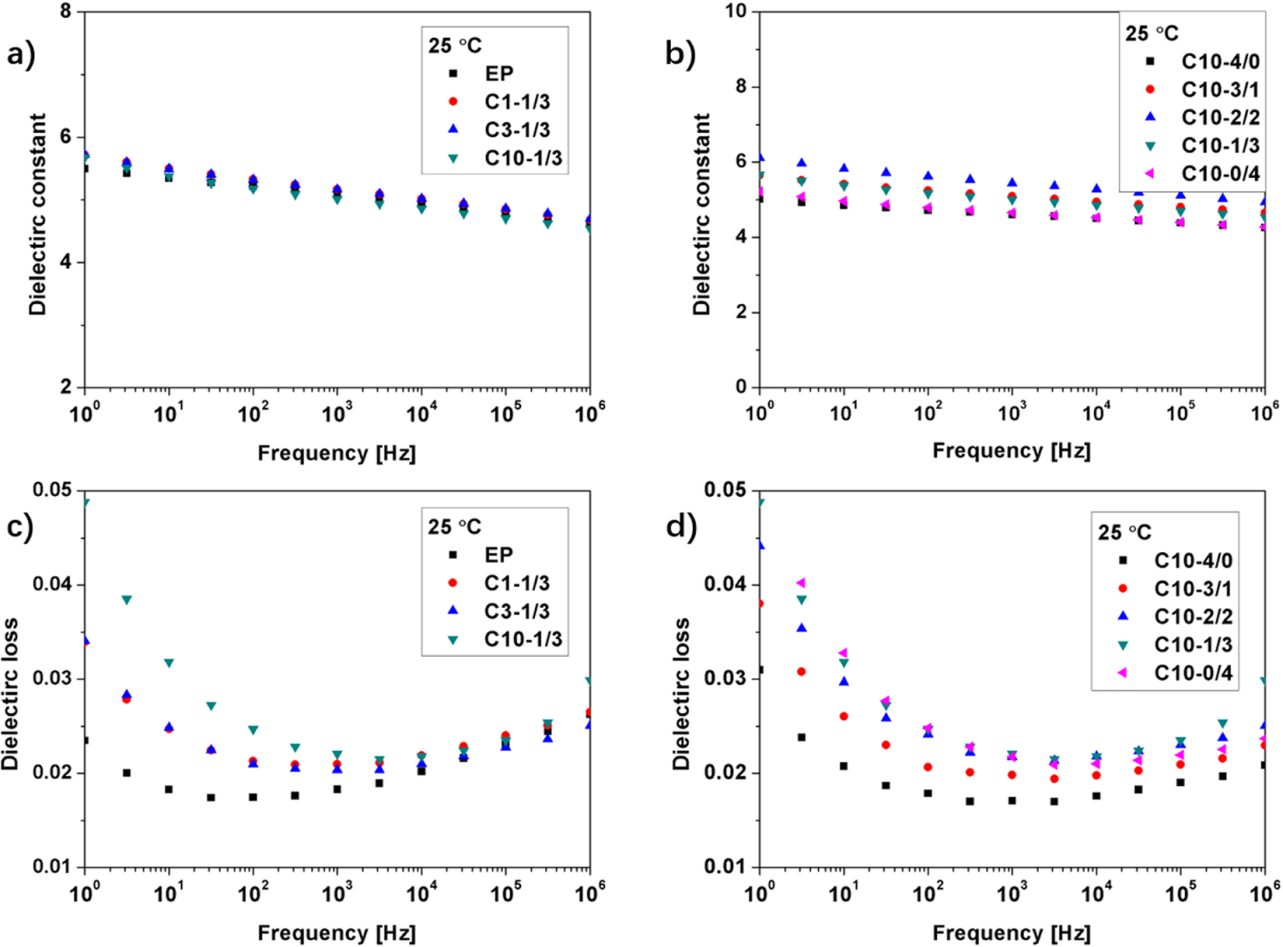
The dielectric constants of the epoxy/SiO2 composites with (**a**) different total SiO2 concentrations and (**b**) different micro/nano proportions. The dielectric losses of the epoxy/SiO2 composites with (**c**) different total SiO2 concentrations and (**d**) different micro/nano proportions.

Dielectric parameters are measured to characterize the interfaces in the different composites. For all the composites, the dielectric constants decrease with increasing frequency as shown in Fig. 3a and b, which is mainly due to the dielectric relaxation. Because the dielectric constants of SiO2 and this epoxy resin are ~4 and ~5, the dielectric constants of all the composites are close to each other. For example, at 1 kHz, all the dielectric constants are within 5.0 ± 0.5.

The differences among the curves of dielectric losses become smaller with increasing frequency. Figure 3c shows that the dielectric losses of CX-1/3 increase with the increase of SiO2 concentration, e.g., from 0.0175 of EP to 0.0247 of C10-1/3 at 100 Hz. Figure 3d shows that the dielectric losses of C10-X/X increase with the increase of the nano SiO2 proportion, e.g., from 0.0179 of C10-4/0 to 0.0248 of C10-0/4 at 100 Hz. The dielectric parameters of the composites are affected by the dielectric polarization and relaxation. In this case, these are polarizations associated with epoxy as well as SiO2 particles and interfacial polarizations at the polymer-particle interfaces^34–36^. It can be inferred that higher SiO2 concentration and higher nano SiO2 proportion introduce more polymer-particle interfaces into the composites.

## Discussion

### Electrical tree growth in the epoxy/SiO2 composites

Since the electrical tree can hardly be observed clearly in the composites with SiO2 concentrations higher than 1 wt% in electrical tree breakdown tests. The electrical tree growth of the composites with low SiO2 concentrations are studied in order to further investigate the effects of the micro SiO2 on resistance to electrical tree. To avoid the quick breakdown of the samples, the distance between the needle electrode and the grounding surface is set as 2 mm rather than 1 mm used in the electrical tree breakdown tests.

**Figure 4:**
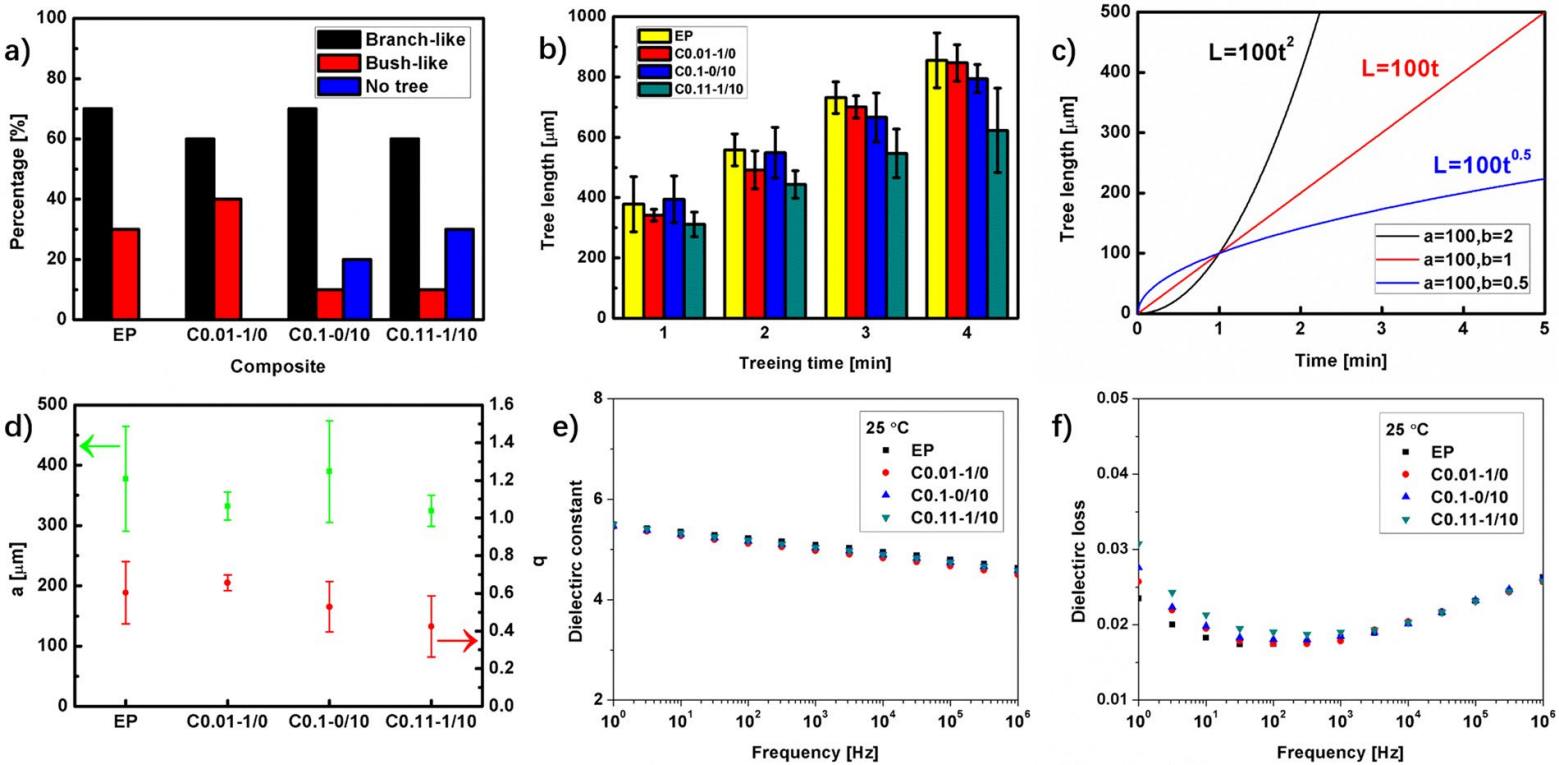
(**a**) The percentages of the electrical tree shapes: branch-like, bush-like and no tree. (**b**) The average tree length of the composites in first 4 min. (**c**) Three patterns of the electrical tree growth. (**d**) The length parameter $a$ and the speed parameter $b$ of the composites. (**e**) The dielectric constants and (**f**) the dielectric losses of the epoxy/SiO2 composites.

According to Fig. 4a, the SiO2 particles does not significantly affect the percentage of branch-like tree, but the nano SiO2 reduces the probability of treeing. As shown in Fig. 4b, after 4 min, the tree length of C0.11-1/10 is 623 um, 27% shorter than 856 um of EP. It can also be found that before 600 um, the tree length of C0.01-1/0 is shorter than that of C0.1-0/10 and becomes longer than C0.1-0/10 after 600 um, which indicates that the micro SiO2 may play a more important role in blocking the electrical tree when the tree is near the needle electrode.

The electrical tree growth can be approximately shown as:

1 $$L=a{t}^{b}$$

where $L$ is the length of the electrical tree, $t$ is the treeing time, $a$ is the length parameter and $b$ is the speed parameter. Figure 4c shows the three possible patterns of the electrical tree growth. When $b$ is 1, the length of the electrical tree grows linearly with the increasing treeing time, If $b$ is larger or smaller than 1, the tree length increases faster or slower as time goes. Most of the treeing patterns in this paper are close to the case when $b\le 1$. The length parameters $a$ and the speed parameters $b$ of the electrical trees in the composites (Supplementary Information 3) are shown in Fig. 4d. It can be seen that the $a$ and the $b$ of C0.11-1/10 are 324 um and 0.424, both of which are the smallest among the four tested composites. Interestingly, it is found that the length parameter $a$ of micro composite is the nearest to that of the micro-nano composite, while the speed parameter $b$ of the nano composite is the nearest to that of the micro-nano composite. These results indicate that after the tree initiation, the micro SiO2 may play a more important role in the early stage of the tree growth (300~400 um) since the value of the length parameter is equal to the tree length after 1 minute, and the nano SiO2 may affect the later stage more by slowing down the treeing speed.

Figure 4e and f show the dielectric constants and the dielectric losses of EP, C0.01-1/0, C0.1-0/10 and C0.11-1/10. The dielectric constants of all the composites are still close to each other. For example, at 1 kHz, all the dielectric constants are within 5.0 ± 0.1. The differences among the curves of dielectric losses become smaller with increasing frequency. The dielectric losses of the composites increase with the incorporation of nano SiO2, e.g., from 0.0175 of EP to 0.0181 of C0.1-0/10 and 0.0190 of C0.11-1/10 at 100 Hz. The increase of the dielectric loss is mainly due to the interfaces introduced by the nano particles.

### Blocking effects of the micro particles

**Figure 5:**
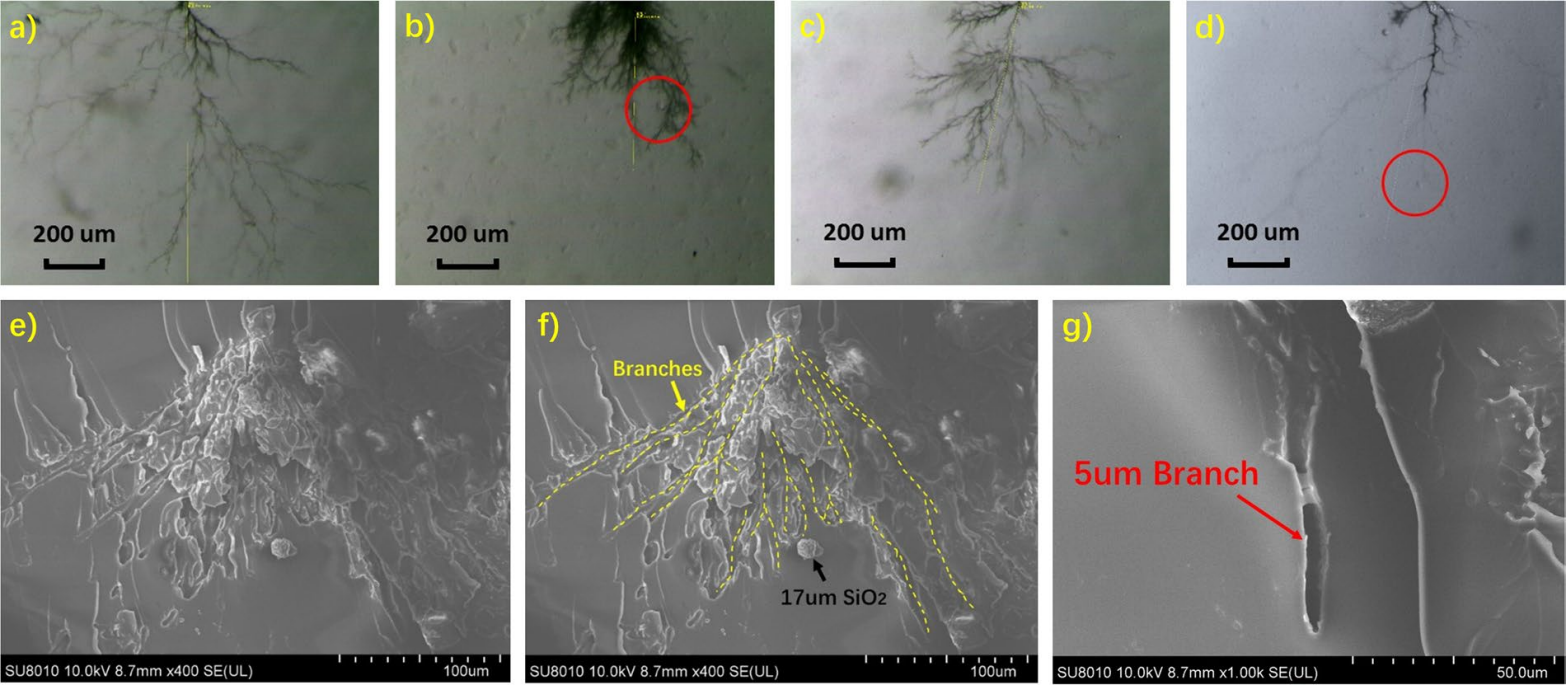
Several typical ways of the electrical tree growth in (**a**) epoxy, (**b**) micro composite, (**c**) nano composite and (**d**) micro-nano composite. SEM images of (**e**), (**f**) an electrical tree with the micro particle and (**g**) a tree branch.

Although a large number of researches have inferred that the particles will hinder the treetop to make the electrical tree turn aside and thus block the electrical tree channel^6, 9, 28, 29, 37^, none of them provides a direct graphic evidence of the blocking effects. As shown in Fig. 5a to d, several typical ways of the electrical tree growth in the different composites are observed under a stereomicroscope.

The polymer degradation will cause the accumulation of the conductive carbon in the electrical tree channel, which makes the channel looks black. As shown in Fig. 5a and c, the electrical trees relatively grow more freely in the neat epoxy and the nano composite. Whereas in the micro composite shown in Fig. 5b, the existence of a 20 um SiO2 particle blocks the 5 um wide tree channel and the tree has to bypass the micro SiO2 particle. This kind of bypass caused by the blocking effects consumes more energy provided from the electrical field. In the micro-nano composite shown in Fig. 5d, the middle tree is blocked by a micro SiO2 particle. Then, the middle tree makes a small turn and just stops growing. Figure 5e gives a SEM image of an electrical tree encountering a 17 um SiO2 particle which obstructs the tree channel. The channels of the tree branches are roughly marked with the yellow dashed lines as shown in Fig. 5f. Figure 5g shows that the width of a tree branch is about 5 um which is smaller than 20 um, the average size of the used micro SiO2. It indicates that the blocking effects of the micro particles are due to their bigger sizes than the tree channels.

The stronger effect of the micro SiO2 on the early stage of the tree growth is probably due to the gradually increasing numbers of the treetops. At the beginning of the tree growth, only several treetops form in a small region near the needle tip. Once the treetops in this small region are influenced by the micro SiO2, the growth of this tree is slowed down. However, at the later stage of the tree growth when treetops are usually much more than the early stage, if the treetops in a certain area are blocked by a micro SiO2 particle, the other treetops which do not encounter micro particles will still grow forward. Actually, when the whole tree is wide enough, the blocking effects of the micro SiO2 particles are very limited. Even if some treetops are blocked by the micro SiO2, the treetops in other regions will gain more power to grow because generally the fewer treetops means the more concentrated electrical energy at the treetops to damage the polymer. It explains why the micro SiO2 plays a more important role in blocking the electrical tree when the particles are near the needle electrode as shown in Fig. 4b and d, which indicates that the micro SiO2 particles affect the electrical tree growth mostly in a structural way.

### Inhibiting effects of the nano particles

The nano particles are believed to inhibit the growth of the electrical tree in many different ways. Nano particles can introduce more submicro-size voids into the polymer matrices and expand the size of the damage process zone of dielectrics^14^, act against partial discharge erosion and expand the electrical tree in diameter in its growing stage^37^, and behave as obstacles to suppress the tree propagation^22^. These all lead to the increasing probability of bush-like tree, which has more treetops for partial discharge and consumes more energy during the tree propagation than branch-like tree. However, in the electrical tree growth tests, the nano particles are not observed to obviously increase the probability of bush-like tree but the probability of no tree. The measurement results of the dielectric parameters indicate that the polymer-particle interfaces may be the dominant factor to inhibit the electrical tree inception and propagation.

The change of the dielectric loss is induced by the interfacial polarizations in the composites^32, 33^. When the real part of the permittivity $\varepsilon^{\prime}$ (dielectric constant) is almost unchanged, the dielectric loss $\tan \delta$ is dominated by the imaginary part of the permittivity $\varepsilon^{\prime\prime}$ which is related to the electrical conductivity.

In Debye polarization model, the $\varepsilon^{\prime\prime}$ can be described as^38^:

2 $$\varepsilon ^{\prime\prime} (\omega )=\frac{A\omega R{C}^{2}}{1+{(\omega RC)}^{2}}\,$$

where $\omega$ is the angular frequency, $A$ is a constant determined by the shape parameters of the dielectric, $R$ is the equivalent resistance and $C$ is the equivalent capacitance. At the frequency higher than 1 Hz, when $\omega {RC}\gg 1$, equation (2) can be written as:

3 $$\varepsilon ^{\prime\prime} (\omega )=\frac{A}{\omega R}$$

As for the dielectrics, the resistance decreases with the increasing temperature. Taking temperature into consideration^39^:

4 $$\varepsilon ^{\prime\prime} (\omega ,T)=\frac{A}{\omega {R}_{0}\exp (\frac{W}{kT})}$$

where $T$ is the temperature, ${R}_{0}$ is the equivalent resistance at extremely high temperature, $W$ is the active energy and $k$ is the Boltzmann constant.

At the same concentration, the interface area of the nano SiO2 is around 1000 times the micro SiO2, more interfaces lead to more interfacial polarizations which will cause electric double layers and inhibit the charges injected from the needle electrode^16, 17^. The electric double layers formed at the polymer-particle interfaces contain mobile charges^9^ and thus increase the conductivity of the interface zones. As a result, the ${R}_{0}$ of the nano composites will be lower than that of the micro composites and thus nano composites have a higher $\varepsilon^{\prime\prime}$ and $\tan \delta$ shown in Fig. 3d.

**Figure 6:**
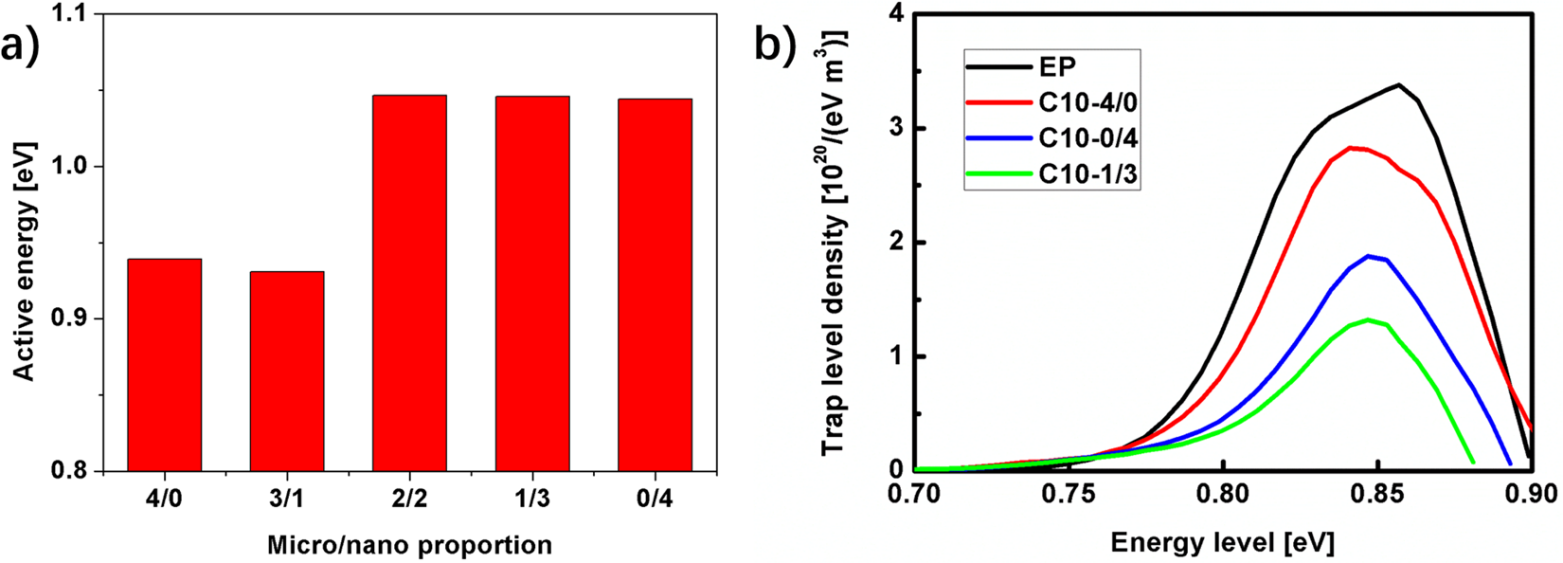
(**a**) The active energy of epoxy/SiO2 composites with different micro/nano proportions at 10 wt%. (**b**) The trap level density distribution of EP, C10-4/0, C10-1/3 and C10-0/4.

Equation (4) can be also written as:

5 $$\mathrm{log}({\varepsilon }^{^{\prime\prime} })=-\mathrm{log}(\omega )+\,\mathrm{log}(A)-\,\mathrm{log}({R}_{0})-\frac{W}{kT}\,\mathrm{log}(e)$$

where $e\,\approx \,2.718$. The active energy $W$ can be calculated from the parallel parts of the curves at low frequencies under different temperatures (Supplementary Information 4). As shown in Fig. 6a, the $W$ of C10-4/0 and C10-3/1 are much lower than those of C10-2/2, C10-1/3 and C10-0/4. For example, the $W$ of C10-4/0 is 0.939 eV and the $W$ of C10-0/4 is 1.044 eV. These results show that the active energy of the nano composites is higher than the micro composites, so the change of the dielectric loss is less sensitive to the increase of the temperature (Supplementary Information 4). Therefore, the higher active energy indicates that the nano composites also need more energy to decrease the equivalent resistance, which aslo means the more energy needed to increase the mobility of the charges.

Figure 6b shows the trap level density distribution of the composites obtained by numerical calculation method^40^ according to the thermally stimulated current (TSC) curves. The incorporation of nano particles decreases the trap level density of the composite, which is similar to the results in previous research^41^. Because the origin of depolarization current is the injection of electrons/holes, the less trap level density detected indicates the less injection of charges, which is caused by the interface introduced by the nano particles. The average trap depths of the samples can be estimated through the half-width method^42^, which gives the average trap depths of EP, C10-4/0, C10-1/3 and C10-0/4 as 0.74 eV, 0.77 eV, 0.96 eV and 1.02 eV (Supplementary Information 5). Trapping sites exist in the interfaces between nano SiO2 particles and epoxy polymer matrices. Lewis^43^ described two kinds of trapping. One is at the chain ends, the chain kinks or the crosslinking sites. The other kind is the sites where activation to a higher energy state is necessary to maintain a continuous path in the interfaces along the field direction. From the results of trap depths, it can be inferred that nano SiO2 introduce the second kind of traps into the composite by their interfaces with polymer matrices and this kind of traps is deeper than the first kind in epoxy. The deeper trap depths introduced by the nano particles will hinder the movement and the transport of the charge carriers, which contributes to the enhancing the stability of polymer against the attack of the charge carriers.

Shimizu’s tree initiation theory^44^ indicates that under AC voltage, the charge injection and extraction break the bonding of the polymer and lead to the tree initiation. Repeated charge injection and extraction may cause substance degradation and electron avalanche breakdown to form the tree channels. The electron avalanche is affected by the energy and the mobility of the electrons. In the nano composites, the electrons will be affected by the nano particles and the electron avalanche is restrained^21^. It is believed that tree initiation must be influenced by some potential barrier formed near the tip of the needle electrode^37^. This barrier is connected to the field formed around nano particles. Later researches pointed out that this potential barrier may be due to the homocharges trapped around needle tip^19, 20^. The equivalent needle tip radius is increased by these homocharges, so the actual electric field strength near needle tip is mitigated and the voltage required for charge injection is raised. In this case, the mobility of the electrons decrease and the electrons gain insufficient energy. Then it will be hard for electron avalanche to form and this will therefore lower the tree inception probability. Tanaka^45^ indicated that high energy electrons must not interact directly with nano particles, but with electronic states in the interfaces between nano particles and polymer matrices. The travelling electrons are affected by these electronic states and shown as trapping. The deeper traps are more likely to play a role in the interaction because of their trapping capability. They can decelerate electrons to retard the formation of tree channels.

It can be concluded that the inhibition of the charges injection and movement will reduce the probability of breaking the molecular chains which causes the polymer degradation, the electrical tree inception and the electrical tree growth. The interfacial polarizations and traps are all directly related to the polymer-particle interfaces in the nano composites. As a result, the inhibition of the electrical tree inception shown in Fig. 4e and the tree growth shown in Fig. 4f is contributed to the large number of the interfaces in the nano composites.

### Synergistic effects of the micro and nano particles

Both the blocking effects of the micro particles and the inhibiting effects of the nano particles have been demonstrated in the micro-nano composites. It should be pointed out that these two effects are not linear combined due to the composite structure of micro particle covered by nano particles. As shown in Fig. 1, the micro particle is covered with the nano particles, which will significantly increase the interface area of the micro particle region. When the electrical tree reaches the micro particle, it has to turn because of the blocking effects. Then, the tree grows along the normal electrical field which is weaker than the tangentiale lectric field and is more easily to be temporally stopped by the inhibiting effects of the nano particle interfaces around the micro particle. The resistance caused by this process can be called as the synergistic effects of the micro and nano particles.

It should be noticed that with this kind of composite structure of the micro and nano particles, the resistance to the electrical tree is stronger when the nano particles are more than the micro particles theoretically. The micro/nano proportion is set to be $1{\rm{:}}t$. The volume of the micro particles per unit volume ${p}_{m}$ is:

6 $${p}_{m}=\frac{1}{1+t}\frac{{V}_{{\rm{SiO}}2}}{V}=\frac{1}{1+t}vol \% $$

where ${V}_{\mathrm{SiO}2}$ and $V$ are the total volume of the SiO2 particles and epoxy/SiO2 composites and ${vol} \%$ is the volume fraction of the total SiO2 particles. The resistance due to the blocking effects of the micro particles ${R}_{m}$ is:

7 $${R}_{m}={K}_{m}\,\sqrt[3]{{{p}_{m}}^{N}}={K}_{m}\,\sqrt[3]{{(\frac{1}{1+t}vol \% )}^{N}}$$

where ${K}_{m}$ is a proportionality constant and $N$ is the dimension of the blocking. The interface volume of the nano particles per unit volume ${p}_{n}$ is:

8 $${p}_{n}=\frac{t}{1+t}\frac{{V}_{{\rm{SiO}}2}}{V}\frac{\frac{4}{3}\pi [{(r+d)}^{3}-{r}^{3}]}{\frac{4}{3}\pi {r}^{3}}\approx \frac{7t}{1+t}vol \% $$

where $r$ is the radius of the nano particle and $d$ is the thickness of the interface. In general, $d\approx r$ ^13^ $.$ The resistance due to the inhibiting effects of the nano particles ${R}_{n}$ is:

9 $${R}_{n}={K}_{n}{p}_{n}={K}_{n}\frac{7t}{1+t}vol \% $$

where ${K}_{n}$ is a proportionality constant. The resistance to the electrical tree $R$ can be defined as the enhancement of the breakdown time:

10 $$R=\frac{t-{t}_{0}}{{t}_{0}}$$

where $t$ is the breakdown time of the composite, ${t}_{0}$ is the breakdown time of the original polymer. When the resistances due to the micro and nano particles are ${R}_{m}$ and ${R}_{n}$, the resistance of the micro-nano composite ${R}_{{mn}}$ should be:

11 $${R}_{mn}=(1+{R}_{m})(1+{R}_{n})-1={R}_{m}+{R}_{n}+{R}_{m}{R}_{n}$$

where ${R}_{m}{R}_{n}$ represents the synergistic effects of the micro particles and the nano particles caused by the composite structure of the micro and nano particles. There are three situations of the resistance to the electrical tree: the electrical trees blocked by the micro particles shown as ${R}_{m}$, the electrical trees inhibited by the nano particles shown as ${R}_{n}$, and the electrical trees inhibited by the nano particles after bypassing the micro particles shown as ${R}_{m}{R}_{n}$. For the composite structure of the micro and nano particles, ${R}_{m}$ and ${R}_{n}$ can be neglected:

12 $${R}_{mn}={R}_{m}{R}_{n}=7{K}_{m}{K}_{n}{(vol \% )}^{\frac{N+3}{3}}\frac{t}{{(1+t)}^{\frac{N+3}{3}}}={K}_{mn}\frac{t}{{(1+t)}^{\frac{N+3}{3}}}\,$$

where ${K}_{{mn}}$ is a proportionality constant related to the total SiO2 concentration. When $t$ is $\frac{3}{N}$, ${R}_{{mn}}$ reaches the maximum.

When the electrical tree reaches a micro particle, the particle act as an obstacle at only two dimensions. The blocking effects do not work at the dimension along the electrical field but at the two dimensions vertical to the electrical field. Therefore, the dimension of the blocking $N$ should be 2. However, the samples used in the electrical treeing experiments are usually platy in order to observe electrical trees under microscope. In this case, these two dimensions are not strictly equal. The electrical trees are restricted in a platy sample so the trees grow more likely in one dimension than the other. The actual value of $N$ should be between 1 and 2, which gives the best $t$ as 1.5 ~ 3. It is approximately consistent with the results shown in Fig. 2f.

## Conclusions

It is shown in the photograph to prove the blocking effects of the micro particles because of the bigger scale than the tree channel. It can be concluded that the inhibiting effects of the nano particles are due to the large number of polymer-particle interfaces which inhibit the charges injection and movement by enhancing the interfacial polarizations and increasing the polymer active energy and the average trap depth. Thus, both incorporation of the micro particles and the nano particles will increase the resistance to electrical tree. It has been demonstrated that the difference in resistance to electrical tree of the micro-nano composites is due to the synergistic effects of the composite structure of the micro and nano particles, which not only reduce the chance of tree inception but extend the electrical tree breakdown time. This research provides a strategy to improve the durability and reliability of polymer dielectrics and extend the lifetime of electrical equipment and electronic devices. The proposed synergistic effects of the micro and nano particles shall exist in other performances of mico-nano composites and be applicable for other types of particles and polymers. Therefore, the new micro-nano composites will find a wide application in future electronic and electrical energy areas.

## Methods

### Materials and Modification

Micro SiO2 (20 um) and nano SiO2 (30 nm) were purchased from Beijing DK nano technology Co., LTD (Beijing, China) and then modified with silane coupling agent KH-550 purchased from Kunshan Lvxun electronic material Co., LTD (Jiangsu, China). 0.6 g KH-550 was dissolved in 200 ml 95% alcohol under stirring for 5 min, then 6 g micro SiO2 or nano SiO2 were added into the solution and the mixture was stirred under room temperature for 2 h. Finally after the slurry was dried at 70 °C for 24 h and 130 °C for 12 h, the KH-550 modified SiO2 micro particles and nano particles were obtained.

### Sample Preparation

Epoxy/SiO2 composites were prepared by solution blending method. The SiO2 particles were first dispersed in chloroform and treated with ultrasound for 3 min at room temperature before the sample preparation. For each composite, a predetermined amount of SiO2 and epoxy were dissolved in chloroform. After stirring for 5 min, the mixture was degassed for 30 minutes at 40 °C and then cured for 2 h at 80 °C followed by 12 h at 100 °C according to a standard curing procedure for this epoxy system. The synthesized composite films were prepared for the dielectric parameters measurement. The cubic samples with pre-embedded steel needle electrode were prepared by curing and sized by a tetrafluoroethylene mould. A piece of conductive rubber was penetrated by the needle electrode and embedded at one side of the sample to provide a conductive contact with the external electrode. The tip of the needle electrode was located near the other side of the 30 × 30 × 3 mm cubic sample (1 mm for electrical tree breakdown tests or 2 mm for electrical tree growth tests from the grounding surface).

### Electrical tests

The electrical treeing experiments were performed with alternating voltage, the samples were clamped between a high voltage plate electrode and a ground plate electrode which were in good contact with the needle electrode and grounding surface of the samples, respectively, and AC high voltage of 15 kV (rms value) was applied to the samples. The development of the electrical tree was observed under a stereomicroscope at 100× magnification. The dielectric constants and the dielectric losses of the composites were measured by a broadband dielectric spectrometer with 1 V from 1 Hz to 1 MHz at 0 °C, 25 °C, 50 °C and 75 °C. The samples were 2 mm thick and had diameters of 20 mm. In TSC tests, the tested samples were first polarized under 300 V/mm at 25 °C for 30 min then cooled down to −20 °C in the speed of −10 °C/min. Then, the samples were depolarized for 10 min to release the polarization charges. Finally, the samples were linearly heated in 3 °C/min with the depolarization current recorded.

## Electronic supplementary material

Supplementary Information
