# Supplementary material for: The Synergistic Effects of the Micro and Nano Particles in Micro-nano Composites on Enhancing the Resistance to Electrical Tree Degradation
Source: Sci Rep. 2017 Aug 17;7:8672. doi: 10.1038/s41598-017-08761-w (PMC5561147; doi:10.1038/s41598-017-08761-w)
Supplement: Supplementary file 1 — Supplementary Information [file 41598_2017_8761_MOESM1_ESM.docx]

**The Synergistic Effects of the Micro and Nano Particles in Micro-nano Composites on Enhancing the Resistance to Electrical Tree Degradation**

Wenxuan Wang, Ying Yang*

*Correspondence to:

yingyang@tsinghua.edu.cn

Department of Electrical Engineering, Tsinghua University, Beijing 100084, China**1. TGA and NMR tests.**

TGA are used to assess the influence of the SiO2 particles on the thermal stability and charring of the composite epoxy resin. TGA tests were carried out at the heating rate of 10 °C per min under nitrogen atmosphere from the room temperature (about 25 °C) to 700 °C.

As shown in Figure S1a and S1b, the early decomposition of the micro molecules begins at 100 °C in EP and composites. All the degradation temperature of the samples is 320 °C, which indicates that the incorporated SiO2 particles do not significantly affect the thermal stability. The increased remaining weight after 400 °C shown in Figure S1a represents the higher incorporated amount of SiO2. The SiO2 particles are modified with the silane coupling agent KH-550 to obtain a better compatibility with the epoxy resin. The sharp decrease before 100 °C is due to the absorbed water from air. Figure S1c indicates that around 5% absorbed water and 7 % attached KH-550 is found on the micro and nano SiO2 particles.

For the investigated materials 1H-NMR spectroscopy is performed. The resonant frequency of 1H was 400 MHz. Samples for the investigations were prepared by cryomilling of unfilled and filled SiO2 samples.

As shown in Figure S1d, the NMR spectrums of the composites show that the peaks of the neat epoxy and the composites are at ~0 ppm and ~6 ppm. After zoomed in, Figure S1e indicate that the incorporation of the SiO2 causes a new peak at ~4 ppm.

**
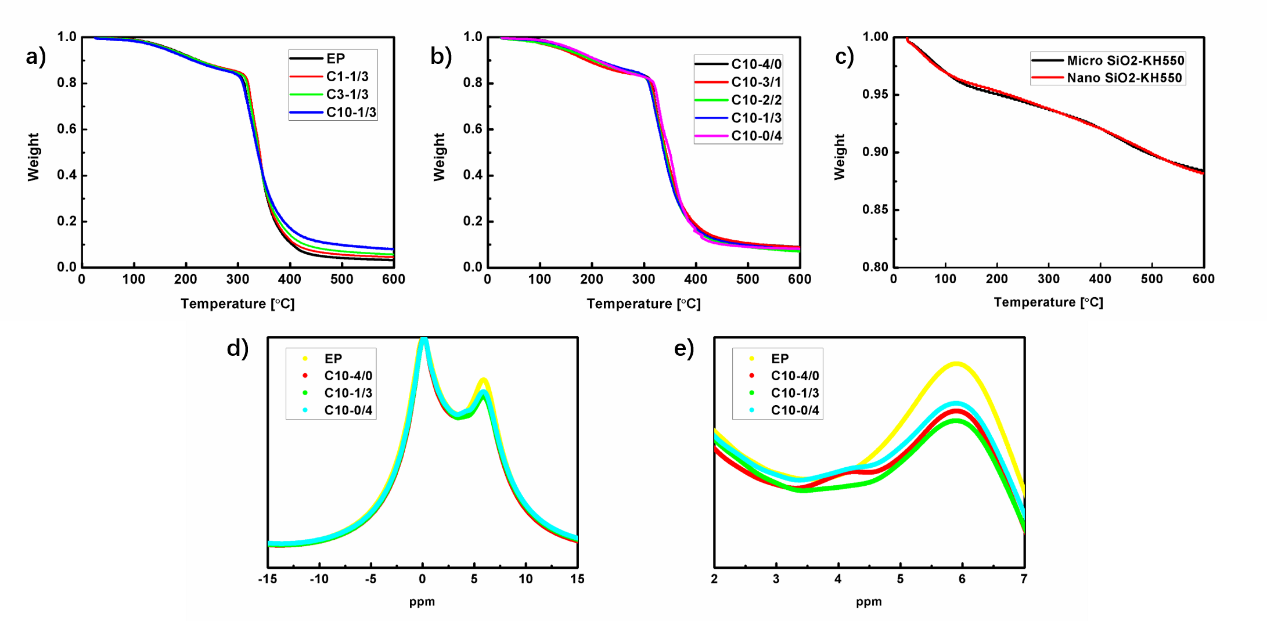
**

**Figure S1.** (a) TGA curves of the epoxy/SiO2 composites with different total SiO2 concentrations. (b) TGA curves of the epoxy/SiO2 composites with different micro/nano proportions. (c) TGA curves of the micro and nano SiO2 modified with the silane coupling agent KH-550. (d)(e) The NMR spectrums of the composites.

**2. Weibull distribution.**

In this research, the conventional two-parameter Weibull function was used to analyze the breakdown time of the composites. The cumulative probability $P$ of the electrical failure time is given by:

$$P\left( t;\alpha,\beta\right)=1-\exp\left\{ -\left( \frac{t}{\alpha} \right)^{\beta} \right\} (1)$$

where $t$ is the breakdown time of the sample, $\beta$ is a shape parameter and $\alpha$ is a scale parameter that indicates the breakdown time at the cumulative probability of 63.2%.

Equation (1) can be also written as:

$$\log\left( -\ln\left( 1-P \right) \right)=\beta\log\left( t \right)- \beta log(\alpha) (2)$$

Thus, the Weibull plot has the relationship shown above between the cumulative probability $P$ and the breakdown time $t$. Several approximations for cumulative probability $P$ have been proposed, the most popular of which is the Ross function and is given by the following equation:

$$P\left( i,n \right)\approx\frac{i-0.44}{n+0.25}\times100\% (3)$$

where $i$ is the $i^{th}$ and $n$ is the total number of each kind of samples. Using Equations (2) and (3), the values of $\beta$ and $\alpha$ for different samples can be obtained.

**3. The growth of the electrical tree.**

Figure S2 show the electrical tree length grows with the increasing treeing time. In general, all the trees of EP reach near 1000 um between 4 and 8 min. As for C0.1-0/10, there is 1 tree does not reach 750 um after 10 min, and no tree is found in 2 samples after 10 min. The micro-nano composite C0.11-1/10 shows a even better resistance to electrical tree: 2 trees do not reach 750 um after 10 min, and no tree is found in 3 samples after 10 min.


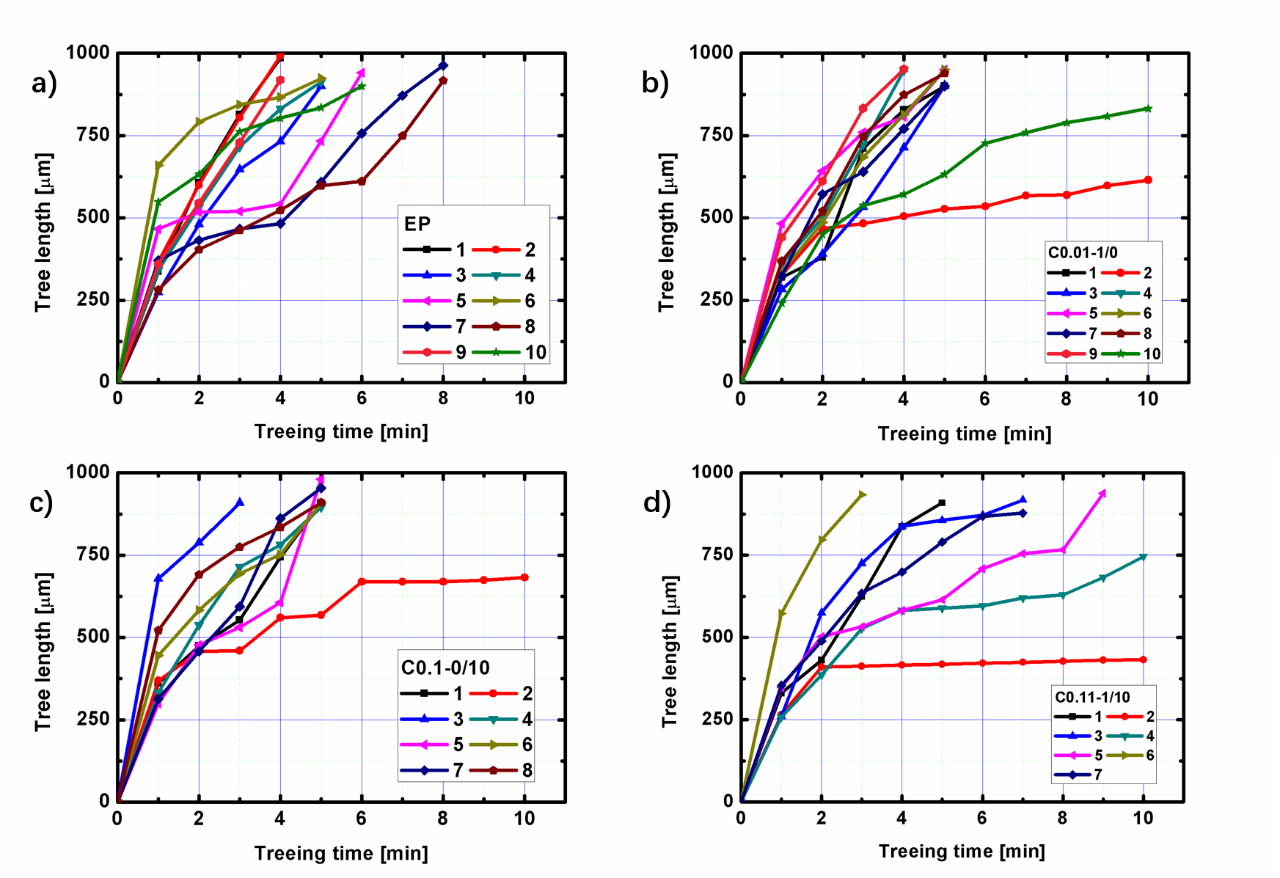
**Figure S2.** (a) ~ (d) The tree lengths of the composites after different treeing time. Each test of one composite is repeated for 10 times. If no tree grows in a sample, then the tree length of this sample will not be plotted.

**4. Dielectric parameters measurement.**

Figure S3 show the $\varepsilon''$ of the composites with different micro/nano proportions under different temperatures for calculating $W$.


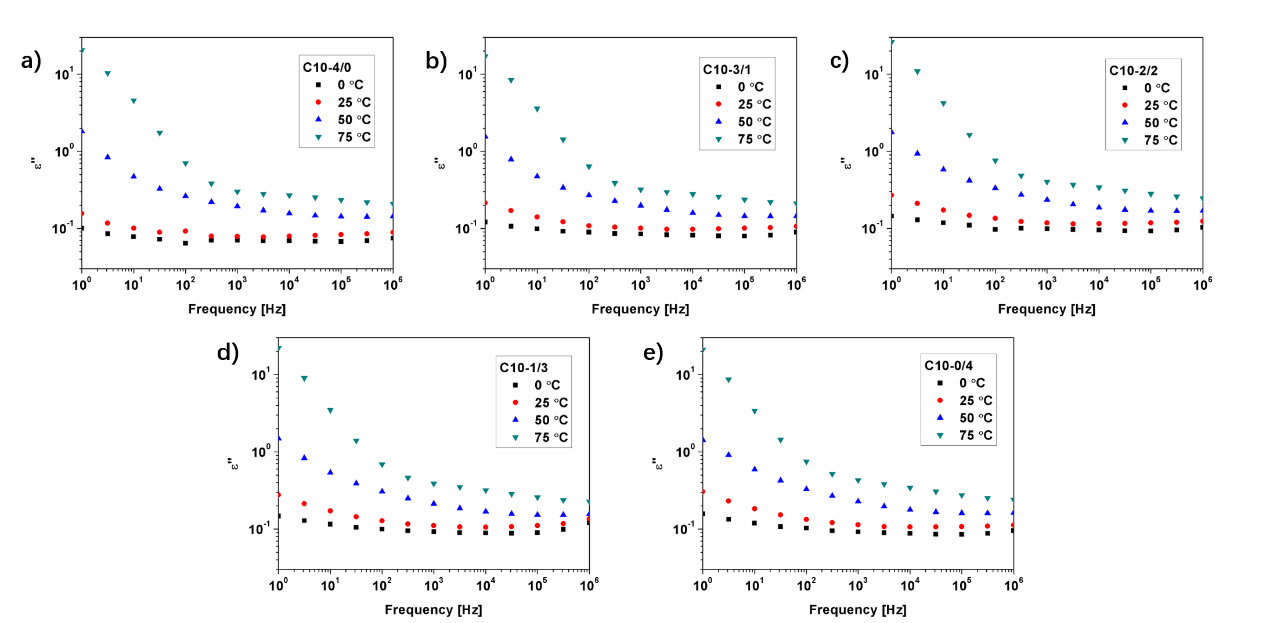


**Figure S3.** The imaginary part of the permittivity of (a) C10-4/0, (b) C10-3/1, (c) C10-2/2, (d) C10-1/3 and (e) C10-0/4.

As shown in Figure S4, the dielectric losses of the composites with higher SiO2 concentration and higher nano SiO2 proportion increase more slowly with increasing temperature, which indicates that higher SiO2 concentration and higher nano SiO2 proportion can lower the sensitivity of dielectric loss to the temperature.


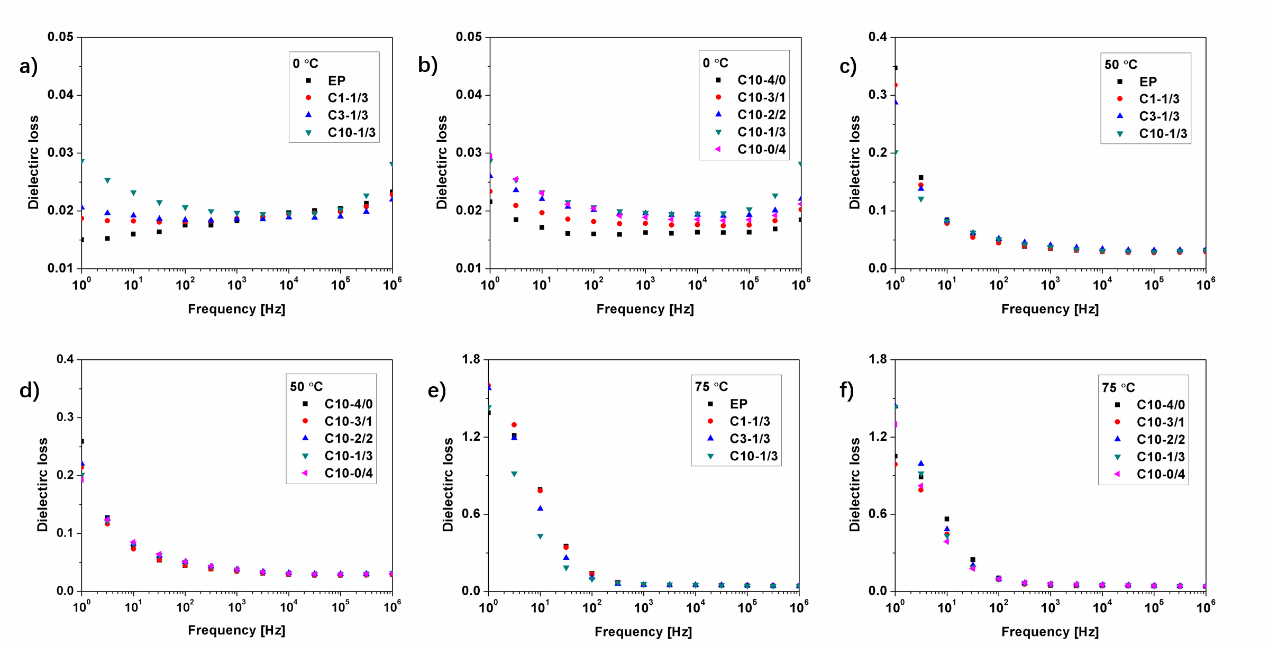


**Figure S4.** The dielectric losses of the epoxy/SiO2 composites with different total SiO2 concentrations and different micro/nano proportions under (a)(b) 0 °C, (c)(d) 50 °C and (e)(f) 75 °C.

**5. The average trap depths obtained from TSC curves** **through the half-width method.**

In order to illustrate the energy level changes of the different samples, the average trap depths of the samples can be estimated through the half-width method:

$$E=\frac{2.47{T_{m}}^{2}k}{\Delta T} (4)$$

where $E$ is the average trap depth, $k$ is the Boltzmann constant, $T_{m}$ is the temperature corresponding to the peak value of TSC curves and $\Delta T$ is the width of the current curve at half peak.


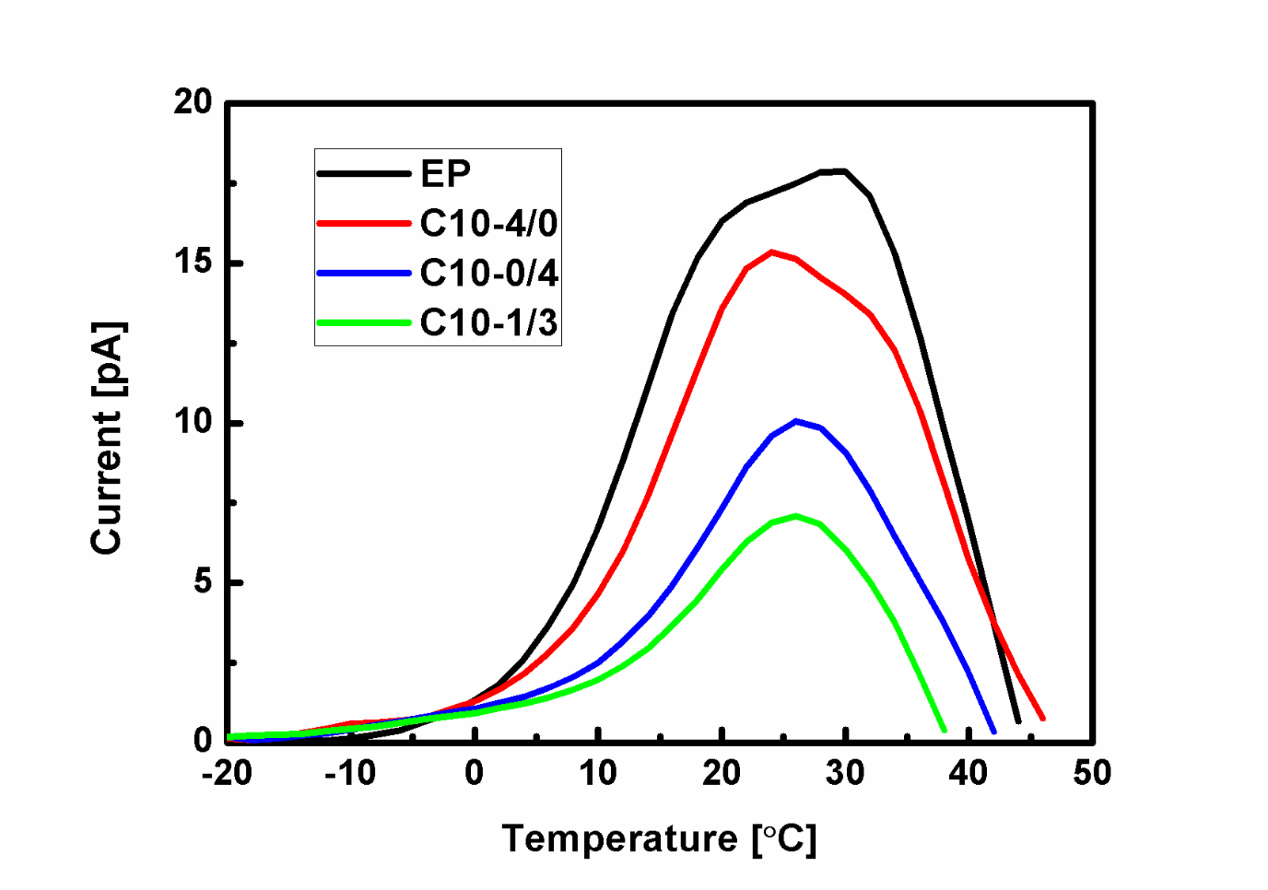


**Figure S5.** The TSC curves of the EP, C10-4/0, C10-0/4 and C10-1/3.

According to Figure S5, the $T_{m}$ and $\Delta T$ can be obtained as shown in Table S1 and the average trap depths $E$ can be calculated.

**Table S1.** The average trap depths of the EP, C10-4/0, C10-0/4 and C10-1/3.

|  | $T_{1}$(^o^C) | $T_{m}$(^o^C) | $T_{2}$(^o^C) | $\Delta T$(K) | $E$(eV) |
| --- | --- | --- | --- | --- | --- |
| EP | 12.1 | 30.0 | 38.6 | 26.5 | 0.74 |
| C10-4/0 | 14.0 | 24.0 | 38.4 | 24.4 | 0.77 |
| C10-0/4 | 16.2 | 26.0 | 36.1 | 19.9 | 0.96 |
| C10-1/3 | 15.7 | 26.0 | 34.3 | 18.6 | 1.02 |
